# Rai14 (Retinoic Acid Induced Protein 14) Is Involved in Regulating F-Actin Dynamics at the Ectoplasmic Specialization in the Rat Testis*

**DOI:** 10.1371/journal.pone.0060656

**Published:** 2013-04-02

**Authors:** Xiaojing Qian, Dolores D. Mruk, C. Yan Cheng

**Affiliations:** 1 The Mary M. Wohlford Laboratory for Male Contraceptive Research, Center for Biomedical Research, Population Council, New York, New York, United States of America; 2 Department of Anatomy, Histology and Embryology, School of Basic Medicine, Peking Union Medical College, Beijing, China; University Hospital of Münster, Germany

## Abstract

Rai14 (retinoic acid induced protein 14) is an actin binding protein first identified in the liver, highly expressed in the placenta, the testis, and the eye. In the course of studying actin binding proteins that regulate the organization of actin filament bundles in the ectoplasmic specialization (ES), a testis-specific actin-rich adherens junction (AJ) type, Rai14 was shown to be one of the regulatory proteins at the ES. In the rat testis, Rai14 was found to be expressed by Sertoli and germ cells, structurally associated with actin and an actin cross-linking protein palladin. Its expression was the highest at the ES in the seminiferous epithelium of adult rat testes, most notably at the apical ES at the Sertoli-spermatid interface, and expressed stage-specifically during the epithelial cycle in stage VII-VIII tubules. However, Rai14 was also found at the basal ES near the basement membrane, associated with the blood-testis barrier (BTB) in stage VIII-IX tubules. A knockdown of Rai14 in Sertoli cells cultured *in vitro* by RNAi was found to perturb the Sertoli cell tight junction-permeability function *in vitro*, mediated by a disruption of F-actin, which in turn led to protein mis-localization at the Sertoli cell BTB. When Rai14 in the testis *in vivo* was knockdown by RNAi, defects in spermatid polarity and adhesion, as well as spermatid transport were noted mediated via changes in F-actin organization and mis-localization of proteins at the apical ES. In short, Rai14 is involved in the re-organization of actin filaments in Sertoli cells during the epithelial cycle, participating in conferring spermatid polarity and cell adhesion in the testis.

## Introduction

Ankycorbin (ankyrin repeat- and coiled-coil structure-containing protein) was first purified from rat liver as a 125 kDa actin-binding protein, and then cloned using a mouse cDNA library in 2000 [1]. It contained 6 ankyrin repeats near its N-terminus with two coil-coil domains near its C-terminus and was thus called ankycorbin [1]. The gene encoding the ankycorbin was also independently identified and cloned from the human retinal pigment epithelial cell ARPE-19 in 2001 [2], and designated novel retinal pigment epithelial cell gene (*NORPEG*) (also called Rai14, retinoic acid induced protein 14) since the expression of Norpeg (or Rai14) in retinal pigment epithelium was found to be induced by retinoic acid [2], [3]. Mouse ankycorbin (Norpeg) is a 979-amino acid polypeptide, displaying 91% sequence similarity to the human NORPEG protein [2]. Ankycorbin is expressed in many mammalian tissues, but most predominantly in retina, placenta and the testis [2], [3]. Ankycorbin was also found in the human testis, highly expressed in both fetal and adult testes and spermatozoa in humans [4]. Since Rai14 appears to be a more widely used name in the field including several vendors that produced this antibody, we elected to use Rai14 in this report. While this protein was shown to be an actin-associated protein more than a decade ago, its function in the testis remains largely unknown.

In the seminiferous epithelium of mammalian testes, such as rodents, the most noticeable ultrastructure under electron microscopy is the extensive bundles of actin filaments that lie perpendicular to the apposing plasma membranes of Sertoli cells and elongating/elongated spermatids surrounding the heads of developing spermatids (step 8–19 spermatids) at the testis-specific adherens junction (AJ) known as apical ectoplasmic specialization (ES) [5]–[10]. Besides the apical ES, the same ultrastructure of bundles of actin filaments is also found at the Sertoli-Sertoli cell interface at the blood-testis barrier (BTB) near the basement membrane known as the basal ES [7], [11], [12]. These extensive networks of actin filament bundles at the ES, which in turn are restricted to the Sertoli-spermatid and Sertoli-Sertoli interface also confer the unusual adhesive strengths of the apical and the basal ES [13]–[15]. It was estimated that the apical ES was at least twice as strong as the desmosome [13] even though desmosome such as in the skin is considered to be one of the most strongest adhesive junctions in mammalian tissues [16]–[19]. However, these actin filament bundles must undergo extensive re-organization during the epithelial cycle since they need to “break-down” and “reassemble” during spermatogenesis to accommodate the transport of preleptotene spermatocytes across the BTB as well as the transport of spermatids across the epithelium at spermiogenesis [5], [7], [8]. In short, these actin filament bundles requires re-organization, altering between the “bundled” and “de-bundled” configuration to accommodate the transport of germ cells across the epithelium during the epithelial cycle. While the presence of these actin filament bundles and the network of actin-based cytoskeleton necessary for spermatogenesis are known for five decades since the 1970s, there are no reports in the literature attempting to understand the molecular mechanism(s) underlying these changes until recently. For instance, it is now known that the highly restrictive spatiotemporal expression of Eps8 (epidermal growth factor receptor pathway substrate 8, an actin barbed end capping and bundling protein) [20], Arp3 (actin-related protein 3) of the Arp2/3 protein complex (a regulatory protein that induces branched actin polymerization) [21], drebrin E (an actin-binding protein that recruits Arp3 to the ES) [22], and filamin A (an actin cross-linker and bundling protein) [23] in the seminiferous epithelium are involved in altering the actin filaments from their “bundled” to “de-bundled” configuration [24]. Since Rai14 is an actin binding protein in the liver [1], and it is highly expressed in the human testis [4], we thought it pertinent to perform functional studies to examine the physiological role of this protein in ES function in the rat testis.

## Materials and Methods

### Animals and antibodies

Sprague-Dawley rats were purchased from Charles River Laboratories (Kingston, NY). The use of animals reported herein was approved by The Rockefeller University Institutional Animal Care and Use Committee (Protocol number 12506). Antibodies used in this study were listed in **Table 1**.

**Table 1:** Antibodies used for different experiments in this report*.

Antibody	Host species	Vendor	Catalog Number	Working dilution	
				IB	IF
Rai14	Rabbit	Protein Tech Group	17507-1-AP	1∶750	1∶100 (cells)
Rai14	Goat	Santa Cruz Biotechnology	sc-82262	-	1∶50 (testes)
Arp3	Mouse	Sigma-Aldrich	A5979	1∶3000	1∶200
Eps8	Mouse	BD Biosciences	610143	1∶5000	1∶100
Drebrin E	Rabbit	Abcam	ab11068-50	1∶1000	1∶100
Palladin	Rabbit	Protein Tech Group	10853-1-AP	1∶1000	1∶100
N-Cadherin	Rabbit	Santa Cruz Biotechnology	sc-7939	1∶200	-
N-Cadherin	Mouse	Invitrogen	33-3900	-	1∶100
α-Catenin	Rabbit	Santa Cruz Biotechnology	sc-7894	1∶200	-
β-Catenin	Mouse	Zymed/Invitrogen	13-9700	-	1∶100
β-Catenin	Rabbit	Invitrogen	71-2700	1∶250	1∶100
JAM-C	Rabbit	Invitrogen	40-8900	1∶250	-
β1-Integrin	Rabbit	Santa Cruz Biotechnology	sc-8978	1∶200	-
Laminin-γ3	Rabbit	Cheng Lab	-	-	1∶100
Afadin	Rabbit	Sigma-Aldrich	A0349	1∶500	-
ZO-1	Rabbit	Invitrogen	61-7300	1∶250	-
ZO-1- FITC	Mouse	Invitrogen	33-9111	-	1∶100
Occludin	Rabbit	Invitrogen	71-1500	1∶250	-
JAM-A	Rabbit	Invitrogen	36-1700	1∶250	-
α-Actinin	Goat	Santa Cruz Biotechnology	sc-7453	1∶200	-
α-Actinin	Mouse	Invitrogen	13-9700	-	1∶100
CAR	Rabbit	Santa Cruz Biotechnology	sc-15405	1∶200	-
Vimentin	Mouse	Santa Cruz Biotechnology	sc-6260	1∶300	-
Actin	Goat	Santa Cruz Biotechnology	sc-1616	1∶300	-
GAPDH	Mouse	Abcam	ab8245	1∶2000	-

* , all antibodies used herein cross-reacted with the corresponding proteins in the rat as indicated by the manufacturers. IB, immunoblotting; IF, immunofluorescence microscopy. The anti-laminin-γ3 antibody was prepared in our laboratory and its specificity was characterized as earlier described [46].

### Treatment of adult rats with adjudin, 1-(2,4-dichlorobenzyl)-*1H*-indazole-3-carbohydrazide

A single dose of adjudin (50 mg/kg body weight) was administered to adult rats (280∼300 g body weight) via gavage [20]. Animals were euthanized by CO_2_ asphyxiation at specified time points, testes were removed, snap-frozen in liquid nitrogen and stored at −80°C. Samples from both treatment *versus* control groups were processed simultaneously to avoid inter-experimental variations. Each time point had at least *n* = 3–4 rats including controls.

### Primary testicular cell cultures

Sertoli cells were isolated from testes of 20-day-old rats [25]. Sertoli cells were seeded on Matrigel (BD Biosciences, San Jose, CA) coated (i) coverslips [cell density at 0.04×10^6^ cells/cm^2^, each coverslip was then placed in a well of 12-well dishes and each well contained 2-ml F12/DMEM containing other supplements (e.g., gentamicin, epidermal growth factor, insulin, transferrin, and bacitracin) as described [25]], (ii) 12-well culture dishes (cell density at 0.4×10^6^ cells/cm^2^, each dish contained 2-ml medium) or (iii) Millicell-HA bicameral culture units (diameter, 12-mm; 0.6 cm^2^ effective surface area) (Millipore, Billerica, MA) (cell density at 1.0×10^6^ cells/cm^2^; each unit was placed in a well of 24-well dishes, and the apical and the basal chamber each contained 0.5-ml medium). Each treatment and control group had triplicate slides, dishes or bicameral units. These cell densities were selected based on pilot experiments so that these cells in cultures were suitable to be used for immunofluorescence microscopy, immunoblotting, and transepithelial electrical resistance (TER) measurement to assess the Sertoli TJ-permeability function. All Sertoli cells cultures were incubated at 35°C in a humidified incubator with 95% air/5% CO_2_ (v/v) as described [25]. About 24 hr after its isolation, Sertoli cells were subjected to a 2.5-min hypotonic treatment using 20 mM Tris, pH 7.4 at 22°C to lyse residual germ cells [26]. Cells were then rinsed twice and cultured in fresh F12/DMEM and used for transfection for RNAi at least 24 hr thereafter. These Sertoli cell cultures had negligible contamination of Leydig, germ and/or peritubular myoid cells when specific markers for these cells were assessed either by RT-PCR or immunoblotting [27] with a purity of at least 95%. To obtain lysates or nucleic acid from Sertoli cells for immunoblotting and/or RT-PCR, cells were terminated on day 4 when a functional tight junction barrier had been established. It is noted that these cells established a functional TJ-permeability barrier, and ultrastructures of TJ, basal ES, gap junction and desmosome were also detected by electron microscopy [7], mimicking the Sertoli cell BTB *in vivo* [25]. Furthermore, this *in vitro* system has been widely used by investigators in the field in studying BTB function [28]–[32]. Furthermore, Sertoli cells isolated from 20-day-old rat testes were fully differentiated and ceased to divide [33] under the conditions that were used herein [25] as characterized earlier [34]–[37]. Also, these Sertoli cells were functionally and physiologically indistinguishable from Sertoli cells isolated from adult rat testes [38] using an established procedure of Wright [39], but adult Sertoli cells were contaminated with germ cells and only a purity of ∼85% was achieved [38], [39]. More important, many of the studies conducted using this *in vitro* system to identify proteins that regulate Sertoli cell BTB function have now been reproduced *in vivo* [7], [20], [21], [40]. Thus, this *in vitro* Sertoli cell system was used herein. Germ cells were isolated from adult rat testes using a mechanical procedure and cultured in serum-free F12/DMEM as described [41]. Total germ cells were harvested for lysate preparation or nucleic acid extraction within 16 hr following their isolation with a viability of >95% when assessed by the erythrosine red dye exclusion test [41].

#### Knockdown of RAI14 in primary Sertoli cells cultured *in vitro*

After Sertoli cells cultured for 2 days, cells were transfected with 100 nM non-targeting negative control siRNA duplexes (Catalog No. 4390844, Ambion) or Rai14 specific siRNA duplexes mixture (Catalog NO. J-087785-9: 5′-UCAAUAAGCAGGUGAGCGA-3′, J-087785-10: 5′-UAGAAGACGCAACCGAAUA-3′, J-087785-11: 5′-AUUCCAAGGUGCUGAACGA-3′, J-087785-12: 5′-GUACAAGAGCCCAGCCGAA-3′, Dharmacon) with Ribojuice siRNA transfection reagent (Novagen). After 24 hr, transfection was terminated by rinsing cells with fresh F12/DMEM twice and then cultured in fresh F12/DMEM for 12 hr to allow recovery. Thereafter, cells were transfected with the siRNA duplexes for another 24 hr (second transfection). About 12 hr thereafter, cells were harvested for lysate preparation or dual-labeled immunofluorescence analysis. For fluorescence microscopy, Sertoli cells were co-transfected with 1 nM siGLO red transfection indicator (Catalog# D-001630-02, Dharmacon) beside siRNA duplexes to assess successful transfection. For TER measurement, Sertoli cells cultured on Millicell bicameral units for ∼2-day were transfected with 150 nM siRNA duplexes in both control and Rai14 RNAi groups for 36 hr.

### Knockdown of RAI14 in the testis *in vivo*

For *in vivo* knockdown of Rai14, adult rats (∼280–300 g b.w., *n* = 4 rats) were treated with non-targeting control *versus* Rai14 siRNA duplexes via intra-testicular injection using a 28-gauge needle [40]. Each testis of the same rat received 100 nM of either the non-targeting control or the Rai14-specific siRNA duplexes on day 0 for transfection. siRNA duplexes were suspended in the transfection mix consisted of 7.5 µl Ribojuice siRNA transfection reagent in 192.5 µl Opti-MEM (Invitrogen) in a final volume of ∼200 µl per testis (the volume of each testis was assumed to be ∼1.6 ml to obtain the desired concentration of the siRNA duplexes). On day 1 and day 2, each testis of the rat was transfected under the same conditions and a total of 3 transfections were performed on each testis. Rats were euthanized by CO_2_ asphyxiation on day 3 (*n* = 1 rat) and day 4 (*n* = 3 rats) with similar phenotypes for both time points. Testes were immediately obtained from these rats, snap- frozen in liquid nitrogen and stored at −80°C until used for examination. All samples including treatment and control groups were examined in a single session to avoid inter-experimental variations.

### Immunoblotting and co-immunoprecipitation (Co-IP)

Lysates were obtained from testis, Sertoli cells and germ cells as described [21], [40]. Protein concentrations were quantified by spectrophotometry using the DC protein assay kit (Bio-Rad Laboratories, Hercules, CA). Immunoblotting was performed [20], [21] using antibodies listed in **Table 1**. Equal protein loading was assessed by using either actin or GAPDH. Co-IP was performed essentially as described [42] using 2 µg IgG for the corresponding primary antibody **(see** **Table 1** **)**. Chemiluminescence was performed using a kit prepared in-house [43]. Densitometric analysis was performed using SigmaGel (Version 1.0).

### RNA extraction and RT-PCR

Total RNA obtained from testes, Sertoli and germ cells were treated with RNase-free DNase I (Invitrogen) to eliminate contaminating genomic DNA, reverse transcribed to cDNA with M-MLV reverse transcriptase (Promega), and target cDNA was then amplified by PCR using Go*Taq* DNA polymerase (Promega) with specific primers **(** **Table 2** **)** essentially as earlier described [40]. The authenticity of PCR products were verified by DNA sequencing performed at Genewiz.

**Table 2:** Primers used for PCR.

Gene	GenBank accession number	Primer orientation	Primer sequence	Nucleotide position	Expected size (bp)
Rai14	BC085775.1	Sense	5′-CTGCTCGTCGCAATACAAAA-3′	571–590	310
		Antisense	5′-AGCAAAGCAGTTGAGTGATG-3′	861–880	
S-16	NM_001169146.1	Sense	5′-TCCGCTGCAGTCCGTTCAAGTCTT-3′	87–110	385
		Antisense	5′-GCCAAACTTCTTGGATTCGCAGCG-3′	448–471	

### Dual-labeled immunofluorescence analysis and F-actin staining

Immunofluorescence microscopy was performed as described [20], [21]. Frozen sections of testes at 7-µm (in thickness) were obtained with a cryostat at −21°C, or Sertoli cells cultured on Matrigel-coated coverslips, were fixed with 4% paraformaldehyde (w/v) in PBS for 10 min, and permeabilized in 0.1% Triton X-100 (v/v) in PBS for 10 min. Testis sections or cells were blocked in 1% BSA (w/v) in PBS for 1 hr, followed by an overnight incubation of primary antibodies **(** **Table 1** **)**, and then an 1 hr incubation of Alexa Fluor conjugated secondary antibodies (Invitrogen, Carlsbad, CA; red fluorescence, Alexa Fluor 555; green fluorescence, Alexa Fluor 488) at a dilution of 1∶100 for cell and 1∶250 for testis section. For F-actin staining, sections or cells were incubated with FITC-conjugated phalloidin (Sigma-Aldrich) at a dilution of 1∶70 or together with the secondary antibody for dual-labeled immunofluorescence analysis. All incubations were performed at room temperature. Sections or cells were mounted in Prolong Gold Antifade reagent with DAPI (4′,6-diamidino-2-phenylindole, a cell nucleus dye) (Invitrogen). Fluorescence images were acquired using an Olympus BX61 microscope with the MicroSuite FIVE software (Version 1.224, Olympus Soft Imaging Solutions Corp., Lakewood, CO) and an Olympus DP70 12.5 MPx digital camera (Olympus America, Melville, NY). Brightness/contrast adjustment and image overlay were performed using Adobe Photoshop in Adobe Creative Suite (Version 3.0; Adobe Systems, San Jose, CA).

### Assessment of TJ-permeability barrier *in vitro*

It was shown that after Sertoli cells were cultured for ∼2–3 days on Matrigel-coated bicameral units, a functional TJ-permeability barrier was established [7], [25]. TER across the Sertoli cell epithelium was measured daily (or at specified time points) to quantify the barrier function using a Millicell electrical resistance system (Millipore) as described [25], [40].

### Semi-quantitative analysis on the efficacy of Rai14 knockdown *in vivo* by fluorescence microscopy

To assess the efficacy of Rai14 knockdown *in vivo*, the intensity of Rai14 fluorescence signals in cross-sections of rat testes at stage VI-VIII was quantified using ImageJ 1.45 (U.S. National Institutes of Health, Bethesda, MD, USA; http://rsbweb.nih.gov/ij) in both Rai14 knockdown *vs.* the non-targeting control groups. These stages were selected since Rai14 was mostly expressed at the ES in these tubules. At least 20 stage VI, VII and VIII tubules from each rat were randomly selected, and the Rai14 signal from each treatment was quantified *vs.* the control group with *n* = 4 rats.

### Assessing changes in the status of spermatogenesis following Rai14 knockdown

To assess changes in the status of spermatogenesis following Rai14 knockdown, frozen sections of testes were obtained at 7 µm thickness in a cryostat at −21°C, and nuclei were stained with DAPI. Since Rai14 is an actin cross-linking protein, we anticipated its knockdown would impede the organization of actin filament bundles at the ES. In this study, we focused on the apical ES because studies on the stage-specific and spatiotemporal expression of Rai14 in the seminiferous epithelium indicated that its localization at the apical ES displayed more subtle changes during the epithelial cycle. We assessed two parameters following Rai14 knockdown: (i) loss of spermatid polarity and (ii) defects in spermiation, were assessed by examining ∼300 randomly selected seminiferous tubules from cross-sections of a testis, and a total of 3 rats were examined. A tubule was scored and annotated as defective if it met one of the following criteria: (i) Loss of spermatid polarity – it was defined by the presence of at least 5 spermatids per cross-section of a tubule in which these spermatids displayed a loss of polarity in which their heads no longer pointing toward the basement membrane but at least 90° deviated from the normal orientation as found in control rat testis; (ii) Defects in spermiation – it was defined by the presence of at least 5 elongating/elongated spermatids that were “trapped” within the seminiferous epithelium after spermiation in a stage IX or X tubule. Data shown in **Table 3** were expressed as percentage of defective tubules in testes transfected with Rai14-specific siRNA duplexes *vs.* the corresponding control rats transfected with nontargeting siRNA duplexes.

**Table 3:** Changes in the status of spermatogenesis in the rat testis following a knockdown of Rai14 by RNAi*.

	Loss of spermatid polarity (stage VI-VIII)**	Defects in spermiation (stage IX-X)***
**Rai14 RNAi ** ***vs.*** ** Ctrl RNAi**	16.12±7.65	6.9±1.26

* Approximately 300 seminiferous tubules at specified stages from each rat testis transfected with Rai14 specific siRNA duplexes (Rai14 RNAi) *vs.*testis transfected with non-targeting control siRNA duplexes (Ctrl RNAi) were randomly selected and scored to assess defects in spermatogenesis, and a total of 3 rats from each group were scored. Data were expressed as percentage of tubules at annotated stages having defects in Rai14 RNAi *vs.* Ctrl RNAi rats.

** Tubules at stage VI-early VIII that contained more than 5 spermatids, displaying a defect in polarity in the seminiferous epithelium in which spermatid heads no longer pointing toward the basement membrane but at least 90° deviated from their normal orientation, were scored as a defect in spermatid polarity.

*** Tubules at stage IX-X that contained more than 5 elongating/elongated spermatids that were trapped in the seminiferous epithelium and failed to undergo spermiation at stage VIII were counted as a defect in spermiation.

### Statistical analysis

Each experiment reported herein was repeated 3–5 times, excluding pilot experiments, using different batches of Sertoli cells. For *in vivo* experiments, each time point has ∼3–4 rats including control group. Statistical analysis was performed using GB-STAT software package (Version 7.0; Dynamic Microsystems, Silver Spring, MD). For multiple comparisons (i.e., 3 or more experimental groups), ANOVA was used for multiple comparison to be followed by Dunnett's test against Ctrl (Ctrl either represents sample at time 0, or untreated/vehicle treated samples at a certain time point). Student's *t*-test was used for paired comparison involving only two experimental groups.

## Results

### Rai14 is expressed by Sertoli and germ cells in the rat testis

**Figure 1:**
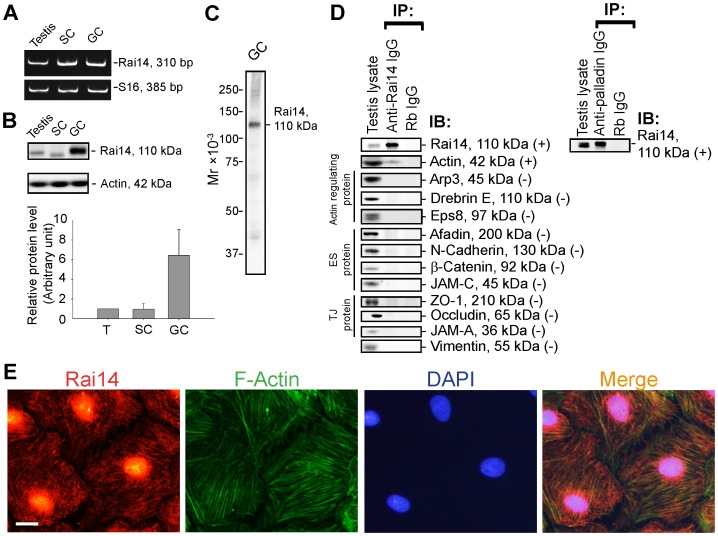
Rai14 is an actin-binding protein in the rat testis. (**A**) A study by RT-PCR to confirm the expression of Rai14 in adult rat testis, Sertoli cells (SC, isolated from 20-day-old rat testes and cultured for 4-day), and germ cells (GC, isolated from adult rat testes and cultured for 16 hr). (**B**) Immunoblotting also confirmed the expression of Rai14 in the rat testis, Sertoli and germ cells, and the relative expression of Rai14 in SC *vs.* GC was shown in the histogram with *n* = 3 experiments in which the relative expression level of Rai14 in the testis was arbitrarily set at 1 so that the relative expression level between these samples can be compared. (**C**) The specificity of the anti-Rai14 antibody (**Table 1**) was assessed by immunoblotting using lysates of GC (20 µg protein). (**D**) Using the specific anti-Rai14 antibody, Rai14 was shown to be an actin-binding protein by co-immunoprecipitation (Co-IP); however, Rai14 did not structurally interact with any of the BTB-associated proteins including several actin-binding and regulatory proteins (*e.g*., Arp3, drebrin E, Eps8) and vimentin (an intermediate filament-based constituent protein). However, Rai14 was found to structurally interact with an actin cross-linking protein palladin which is known to be involved in conferring actin filament bundles in other mammalian cells [48]. (**E**) Rai14 (red) was also shown to be an actin-binding protein by dual-labeled immunofluorescence analysis in which it co-localized with F-actin (green) in Sertoli cells. Cell nuclei (blue) were visualized by DAPI. Scale ba  = 20 µm, which applies to all other micrographs.

Expression of Rai14 by Sertoli and germ cells in the rat testis was confirmed by RT-PCR **(** **Fig. 1A** **)** and immunoblotting **(** **Fig. 1B** **)** using the corresponding specific primer pairs **(** **Table 2** **)** and anti-Rai14 antibody **(** **Table 1** **)**. The specificity of the antibody against Rai14 was also assessed by immunoblotting using lysates of germ cells **(** **Fig. 1C** **)**. Since Rai14 is a known actin-binding protein [1], this anti- Rai14 antibody was used to assess the likely interaction between Rai14 and several actin binding/regulatory proteins earlier found in the testis, as well as ES- and TJ-proteins by Co-IP **(** **Fig. 1D** **)**. Indeed, Rai14 was found to structurally associate with actin and also palladin (an actin cross-linking protein), but none of the other proteins that were examined **(** **Fig. 1D** **)**. Consistent with the finding by Co-IP, Rai14 was found to associate with the actin filaments in Sertoli cells, co-localized, at least in part, with F-actin in Sertoli cells **(** **Fig. 1E** **)**.

### Stage-specific expression of Rai14 at the ES in the rat testis during the epithelial cycle

**Figure 2:**
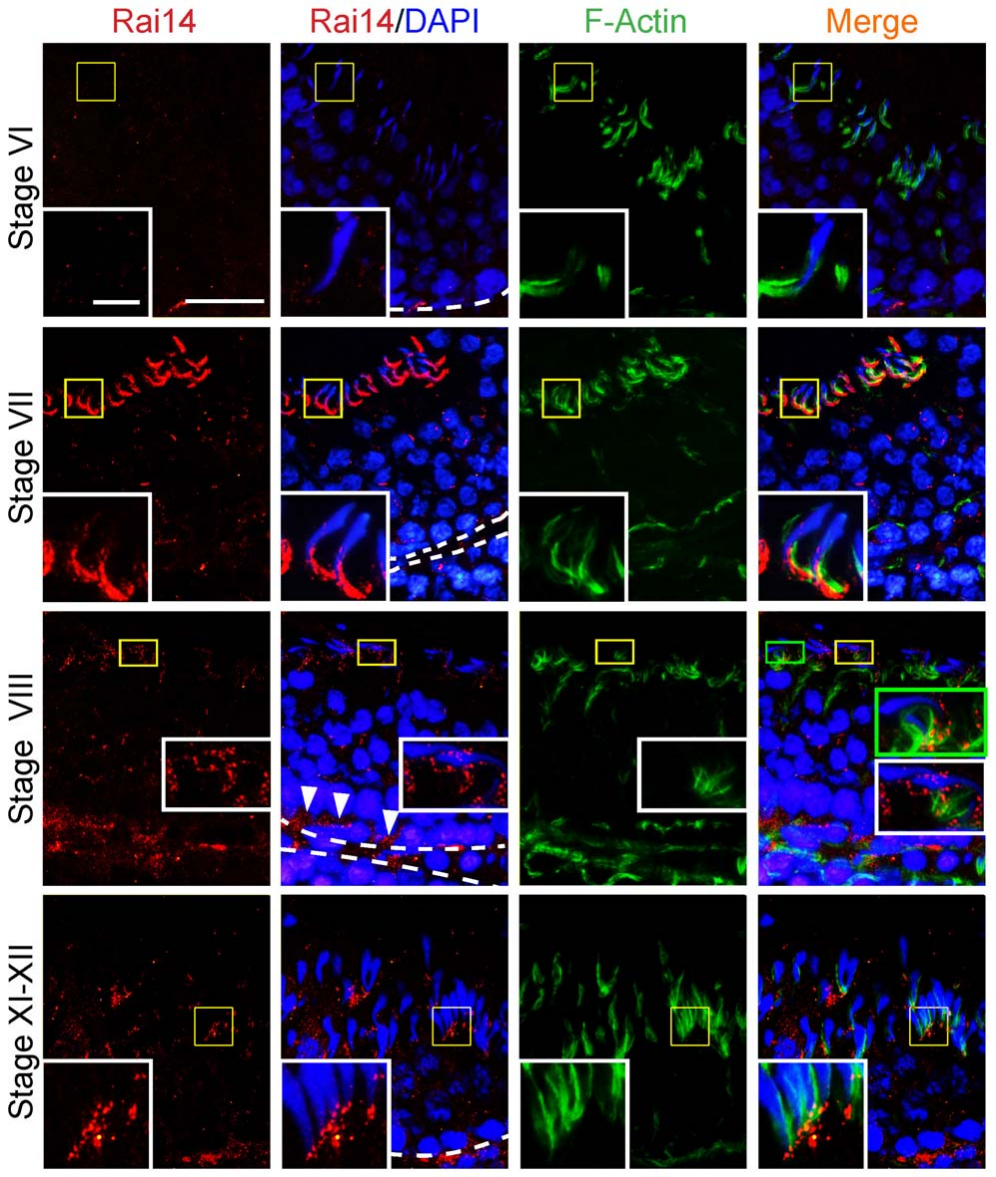
Stage-specific expression of Rai14 and its association with the F-actin-rich ectoplasmic specialization (ES) in the seminiferous epithelium of adult rat testes. Dual-labeled immunofluorescence analysis was performed using frozen cross-sections of testes from adult rat testes to examine co-localization of Rai14 (red) and F-actin (green) in the seminiferous epithelium. At stage VI, Rai14 was weakly detected at the apical ES at the Sertoli-spermatid interface, and its localization to the BTB was also weakly detectable. However, at stage VII-early stage VIII, the expression of Rai14 was the strongest. Rai14 was intensely localized to the apical ES, most abundantly at the front-end of the spermatid head, and co-localized with F-actin at the site (“yellow” in merged image of the apical ES), and its localization at the BTB remained not clearly visible. At stage VIII, Rai14 expression at the apical ES was still considerably strong, but it no longer restricted to the front-end of the spermatid head, instead, Rai14 was scattered around the spermatid head at the apical ES, but not tightly co-localized with the F-actin when compared to late stage VII tubule. In late stage VIII tubules, Rai14 also considerably expressed near the basement membrane (annotated by “white” broken line), consistent with its localization at the BTB (see “white” arrowheads), but not tightly co-localized with F-actin. At stage XI-XII, Rai14 remained localized to the apical ES, but it also shifted to the front-end of the spermatid head, partially co-localized with F-actin. “Yellow” and “green” boxed areas were magnified and shown in corresponding micrographs to better illustrate the localization and/or co-localization of Rai14 and/or Rai14/F-actin at the apical ES. Scale bar = 50 µm or 10 µm in the micrograph or inset, respectively, which apply to all other micrographs and insets.

Using frozen sections of adult rat testes, the expression of Rai14 in the seminiferous epithelium was found to be stage-specific. The localization Rai14 was limited almost exclusively to the ES (an actin-rich AJ specific to the testis), most abundantly at the apical ES but also at the basal ES at the BTB, which is the ultrastructure in the testis that is constituted mostly by bundles of actin filaments that are sandwiched in-between cisternae of endoplasmic reticulum and the apposing Sertoli-spermatid plasma membranes (for the apical ES) or the apposing Sertoli-Sertoli plasma membranes (for the basal ES) [5], [8]. As such, it is not entirely unexpected that this actin binding protein was associated with the apical ES, and the basal ES at the BTB because the maintenance of the ES requires intact actin filament bundles. Rai14 was first detected at the apical ES at stage VI to early VII tubules, surrounding (but diffusely localized) to the entire spermatid head, but it became highly expressed but intensely localized, almost exclusively, to the concave side of the spermatid head and co-localized with F-actin at the apical ES in late stage VII tubules **(** **Fig. 2** **)**. At late stage VIII, the expression of Rai14 considerably diminished, and it was no longer tightly restricted to the concave side of the spermatid head, instead, it was localized surrounding the entire spermatid head, and this pattern of localization persisted through stage XI-XII; and at these stages, Rai14 was not tightly co-localized with actin as of stage VIII **(** **Fig. 2** **)**. Also, Rai14 was also found at the newly formed apical ES that first appeared at the step 8 spermatid-Sertoli cell interface in stage VIII tubules and persisted through step 11–12 spermatids in stage XI-XII tubules **(** **Fig. 2** **)**. While its expression was less abundant near the basal compartment, Rai14 was also found at the tunica propria, possibly associated with peritubular myoid cells, but also at the BTB in stage VIII and stage XI-XII tubules but considerably less at stage VI-VII and stage late VII tubules **(** **Fig. 2** **)**.

### Rai14 partially co-localizes with laminin-γ3, Arp3, drebrin E and palladin at the apical ES, and ZO-1 and N-cadherin at the basal ES

**Figure 3:**
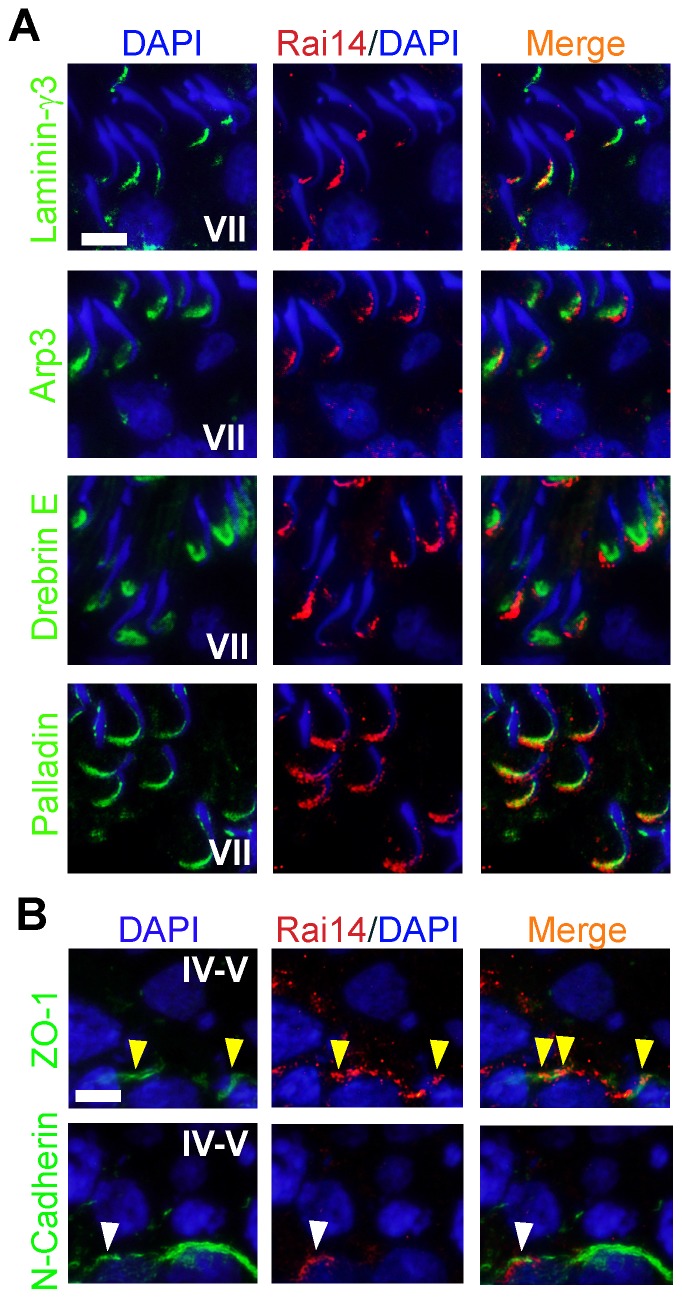
Rai14 is an apical and basal ES protein in the rat testis. Dual-labeled immunofluorescence analysis was used to assess co-localization of Rai14 (red) with constituent protein laminin-γ3 chain (green), actin regulatory protein Arp3 (green), actin binding protein drebrin E (green) and actin filament cross-linking protein palladin (green) at the apical ES in stage VII tubules when these proteins were all highly expressed. It was found that Rai14 indeed partially co-localized with each of these apical ES proteins (see “orange yellow” in merged images) as shown in (**A**). Rai14 (red) also partially co-localized with basal ES/TJ protein ZO-1 (see “yellow” arrowheads), and to a lesser extent with N-cadherin (see “white” arrowheads) in stage IV-V tubules when these proteins were highly expressed as shown in (**B**). Bar = 10 µm in (**A**) or (**B**), which applies all other images in (**A**) and (**B**).

Since Rai14 was shown to be an actin-binding protein when it was first discovered [1] and it was found to associate with the F-actin-rich ultrastructure, namely the apical ES, in the seminiferous epithelium, we next examined if Rai14 co-localized with some of the integrated apical ES component proteins in the rat testis. Laminin-γ3 chain (an apical ES constituent protein restricted to the elongating/elongated spermatids that form a functional ligand with the laminin-α3 and -β3 chains to create an adhesion protein complex with α6β1-integrin which is restricted to the Sertoli cell) [44]–[47], Arp3 (together with Arp2, the Arp2/3 complex confers branched actin polymerization, altering the “bundled” actin filaments to a branched/de-bundled network, destabilizing the apical ES function to facilitate spermatid transport across the seminiferous epithelium during spermiogenesis) [21], drebrin E (an actin-binding protein that has high affinity to Arp3 and shown to recruit Arp3 to the ES to induce actin re-organization) [22] and palladin (a known actin filament cross-linking and bundling protein) [48], [49] were selected in this study **(** **Fig. 3A** **)** because of their involvement in either apical ES dynamics or actin organization at the ES. Indeed, at stage VII when the expression of Rai14 at the apical ES was up-regulated, Rai14 was found to co-localize, at least in part, with laminin-γ3 chain, Arp3, drebrin E and palladin **(** **Fig. 3A** **)**. Furthermore, Rai14 was also co-localized, at least in part, with TJ-adaptor protein ZO-1 and basal ES protein N-cadherin at the basal ES at the BTB in stage IV-V tubules when the expression of these BTB proteins was up-regulated **(** **Fig. 3B** **)**. These findings **(** **Fig. 3A, B** **)** coupled with the continuous changes in its localization at the apical ES during the epithelial cycle **(** **Fig. 2** **)** strongly support the notion that Rai14 is involved in the F-actin organization during the epithelial cycle, most notably at the apical ES.

### Rai14 loses its association with actin at the apical ES during adjudin-induced apical ES disruption that leads to spermatid loss from the epithelium

**Figure 4:**
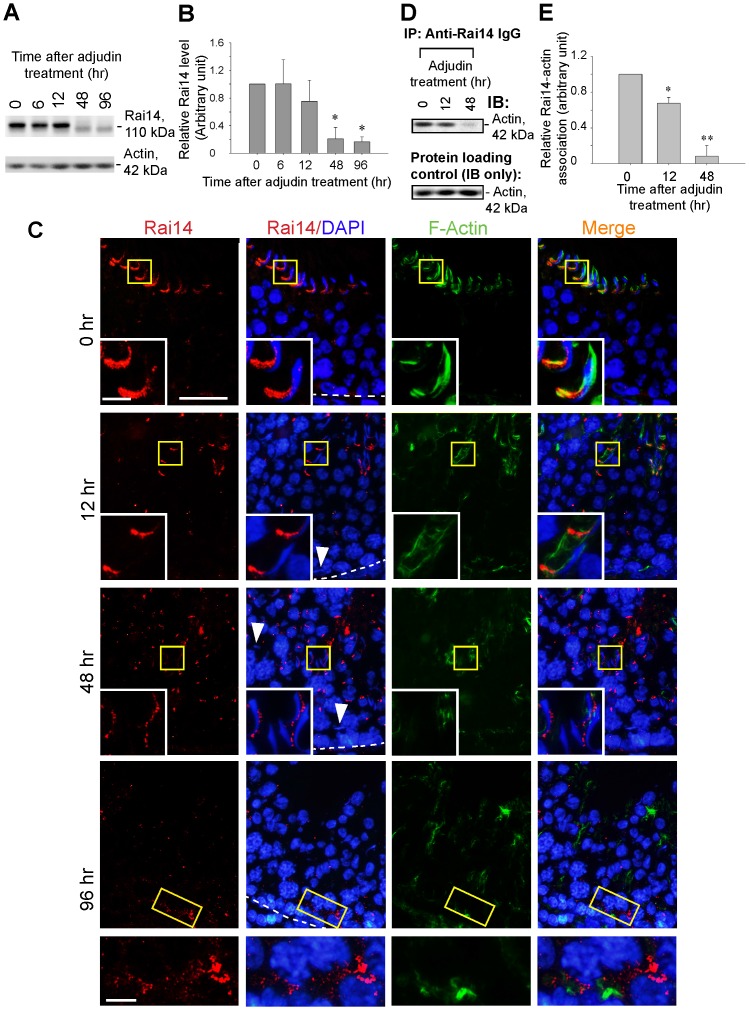
Down-regulation in the expression of Rai14 and a loss of association between Rai14 and F-actin during adjudin-induced apical ES disruption and spermatid loss from the seminiferous epithelium. Adult rats were treated with a single dose of adjudin (50 mg/kg b.w., by gavage) which is known to induce spermatid loss from the seminiferous epithelium since the apical ES, in particular the actin filament bundles at the site, is one of the primary targets of adjudin [7], [24]. Rats (*n* = 3 rats per time point) at specified time points were terminated and used for immunoblotting (A) which illustrated a rapid down-regulation of Rai14 expression following adjudin treatment, and the histogram shown in (B) summarized these findings. Each bar in (B) is a mean±SD of *n* = 3 using immunoblots such as those shown in (A) and normalized again β-actin which served as a protein loading control, and the relative Rai14 level in rats at time 0 (control) was arbitrarily set at 1. *, *P*<0.05. (C) Frozen sections from the testes of these rats were also obtained for dual-labeled immunofluorescence analysis. In control rat testes (0 hr), Rai14 (red) was intensely localized to the apical ES in a late stage VII tubule, localized to the front-end of the spermatid head and co-localized with F-actin (green), and the relative location of the basement membrane was annotated (see “white” broken line). Within 12 hr after adjudin treatment, Rai14 staining at the apical ES in a similar staged tubules was diminished and spermatids failed to migrate across the epithelium, losing their polarity (see “white” arrowhead that annotates a mis-oriented spermatid with its head no longer pointing to the basement membrane and it was “trapped” near the basement membrane without any Rai14 staining); more important, Rai14 no longer co-localized with F-actin, and this trend of diminishing Rai14 expression and loss of co-localization with F-actin persisted through 48 hr (“white” arrowhead also annotates a mis-oriented spermatid and was trapped in the basement membrane) and 96 hr. At 96 hr, expression of Rai14 near the basement membrane, consistent with its localization at the BTB was up-regulated, however, it was not co-localized with the F-actin. Insets in these micrographs are the magnified view of the corresponding “yellow” boxed areas. Bar = 50 µm or 10 µm in the micrograph or in the inset, which applies to all remaining micrographs and insets. (D) A loss of co-localization between Rai14 and F-actin at 12- and 48-hr following adjudin treatment as shown in (C) was further confirmed by Co-IP using testis lysates (800 µg protein) from rats at 0, 12-hr and 48-hr with anti-Rai14 IgG as the precipitating antibody, and the immunoblot was visualized using an anti-actin antibody to assess Rai14-actin interaction (the lower panel is IB only without Co-IP to serve as protein loading control). Data shown in (D) were summarized in (E) with each bar graph  =  a mean±SD of *n* = 3 experiments, a loss in Rai and actin protein-protein interaction was detected by 12-hr (∼35% reduction) and by 48-hr, a loss of ∼90% was detected. *, *P*<0.05; **, *P*<0.01.

To better understand the role of Rai14 on actin dynamics and its involvement in apical ES function, we sought to examine any changes in Rai14 distribution in the seminiferous epithelium during adjudin-induced apical ES restructuring that led to premature release of spermatids from the seminiferous epithelium, mimicking spermiation [24] **(** **Fig. 4A**–**D** **)**. Adjudin [1-(2,4-dichlorobenzyl)-*1H*-indazole-3-carbohydrazide] is a potential male contraceptive [10], [24] that exerts its effects primarily at the apical ES in the rat testis, down-regulating the expression of Eps8 (an actin bundling protein at the ES) [20], inducing mis-localization of Arp3 [21] and drebrin E [22], these changes thus lead to a dis-organization of the actin filament bundles at the apical ES, perturbing apical ES function that causes the premature release of spermatids from the seminiferous epithelium [24]. Interestingly, adjudin was also found to strengthen the BTB integrity by up-regulating the expression of TJ- (*e.g*., occludin) and basal ES (*e.g*., N-cadherin) proteins at the BTB [50], [51], supporting the concept that there is a unique mechanism in the testis to protect the BTB during apical ES disruption, such as at spermiation, since these two F-actin-rich ultrastructures are localized at the opposite ends of the Sertoli cell epithelium [7]. Indeed, a down-regulation of Rai14 expression following adjudin treatment was noted **(** **Fig. 4A, B** **)**. Although, Rai14 was found to co-localize with F-actin at the apical ES in rats in stage VII tubules in control rats, treatment of rats with adjudin (50 mg/kg b.w., via gavage) that induced apical ES disruption was found to cause a loss of spermatid polarity (see “white” arrowhead in which the head of the spermatid no longer pointing toward the basement membrane) and these mis-oriented spermatids were “trapped” in the epithelium near the basement membrane, failing to traverse the epithelium at 12 and 48 hr after adjudin treatment **(** **Fig. 4C** **)**. Also, premature release of spermatids were also noted in which spermatids were gathered in the tubule lumen by 12- and 48-h **(** **Fig. 4C** **)**. Furthermore, a loss of co-localization between Rai14 and F-actin by 12 to 48 hr was also noted **(** **Fig. 4C** **)**. Interestingly, an up-regulated expression of Rai14 was detected at the BTB in tubules virtually devoid of spermatids by 96 hr **(** **Fig. 4C** **)**. This latter finding supports the notion that an increase in Rai14 expression at the BTB may be associated with a tightening of the BTB integrity following adjudin treatment as earlier reported [50]. It is noted that the overall Rai14 expression in the seminiferous epithelium of rat testes following adjudin treatment was down-regulated when examined by immunofluorescence microscopy **(** **Fig. 4C** **)**, consistent with immunoblotting data **(** **Fig. 4A, B** **)**. This loss of association between Rai14 and actin was further confirmed by a Co-IP experiment in which a loss of protein-protein interaction between Rai14 and actin was detected by 12 hr after adjudin treatment and it was more significant by 48 hr **(** **Fig. 4D, E** **)**.

### Rai14 knockdown in Sertoli cells *in vitro* by RNAi perturbs the Sertoli cell TJ-permeability function and perturbs protein distribution at the cell-cell interface

**Figure 5:**
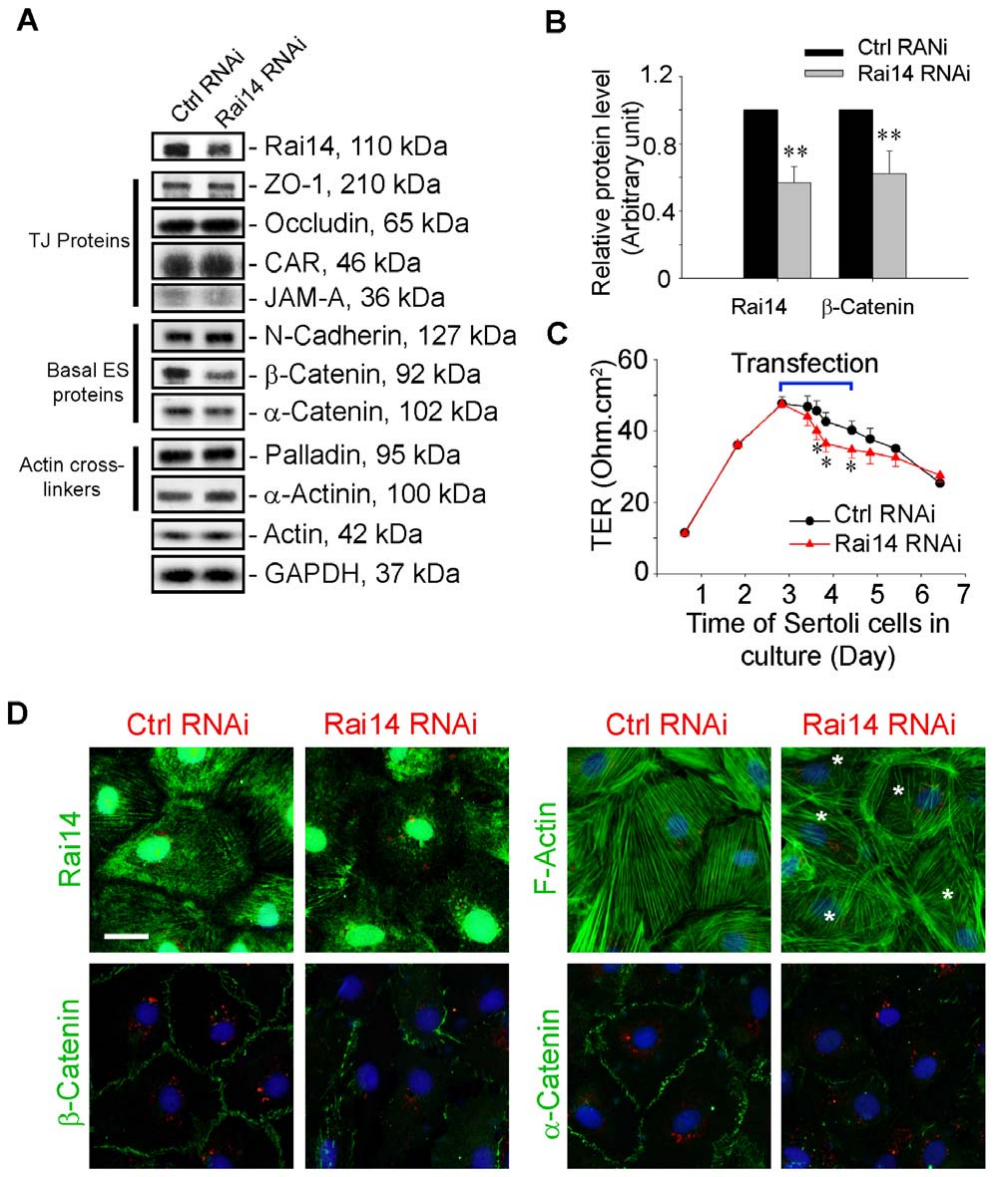
Knockdown of Rai14 in the Sertoli cell epithelium with an established TJ-permeability barrier *in vitro* by RNAi disrupts actin filament organization and the TJ barrier. (A) Sertoli cells cultured alone on Matrigel-coated 12-well dishes for 2-day with an established TJ-permeability barrier were transfected with Rai14 siRNA duplexes (Rai14 RNAi) *versus* non-targeting control duplexes (Ctrl RNAi) at 100 nM using Ribojuice transfection medium for 24 hr, thereafter, cells were washed twice and cultured in F12/DMEM for 12 hr to allow recovery. Thereafter, cells were transfected again under the same conditions for another 24 hr. Thereafter, cells were rinsed with fresh F12/DMEM and cultured for an additional 12 hr before termination, and used to prepare lysates for immunoblotting using antibodies against several BTB-associated constituent or regulatory proteins. A knockdown of Rai14 by ∼50% was noted in which the control was arbitrarily set at 1 against which statistical comparison was performed (B) without any apparent off-target effects (A). The findings shown herein are the results of 3 independent experiments excluding pilot experiments which were used to establish optimal experimental conditions, such as different concentrations of siRNA duplexes and Ribojuice. It was noted that we achieved only ∼50–60% knockdown of Rai14 in several pilot experiments, unlike other target genes [*e.g*., Scribble, β1-integrin, and P-glycoprotein] wherein we could silence the target gene expression by as much as ∼70–90%. **, *P*<0.01. (C) In parallel experiment, Sertoli cells cultured for 2.5-day on Matrigel-coated bicameral units with an established TJ-permeability barrier (manifested by a stable TER across the cell epithelium) were transfected with the Rai14 siRNA duplexes *versus* non-targeting control siRNA duplexes for 36 h, and cells were washed twice and replaced with fresh F12/DMEM. TER was monitored across the Sertoli cell epithelium to assess changes in the TJ-permeability barrier function. Each data point is a mean±SD of triplicate bicameral units, and this experiment was repeated three times using different batches of Sertoli cells and yielded similar results. *, *P*<0.01. (D) Effects of Rai14 on F-actin organization and protein distribution at the Sertoli cell-cell interface were also assessed by immunofluorescence microscopy. The knockdown of Rai14 in Sertoli cells was found to reduce Rai14 staining considerably, consistent with data shown in (A, B). Interestingly, Rai14 knockdown also induced changes in F-actin organization in which the actin filaments were truncated and defragmented in the Sertoli cell cytosol (see asterisks). Furthermore, β- and α-catenin, adaptor proteins at the BTB, were also found to be mis-localized, which no longer localized to the cell-cell interface, but moved into the cell cytosol. Sertoli cells were co-transfected with 1 nM siGLO red transfection indicator to illustrate successful transfection. Scale bar = 20 µm, which applies to all remaining micrographs.

In order to better understand the physiological role of Rai14 in the testis, we next examined if the knockdown of Rai14 by RNAi would impede the Sertoli cell TJ-permeability barrier via changes in the organization of actin filaments in Sertoli cells. Sertoli cells were cultured for ∼2.5 days *in vitro* to establish an intact cell epithelium with a functional TJ-barrier. Thereafter, these cells were transfected with specific Rai14 siRNA duplexes *versus* the non-targeting control duplexes twice for 24 hr each (with a 12-hr recovery in between), and cells were harvested 12 hr later to assess the silencing efficacy by immunoblotting. The knockdown of Rai14 by ∼50% **(** **Fig. 5A, B** **)** did not induce any significant off-target effect when a number of BTB-associated proteins were examined by immunoblotting using corresponding antibodies **(see** **Table 1** **)**. Interestingly, the knockdown of Rai14 by RNAi was shown to perturb the Sertoli cell TJ-permeability barrier transiently **(** **Fig. 5C** **)**, illustrating Rai14 was involved in maintaining the Sertoli cell BTB integrity. To further understand the likely mechanism by which Rai14 regulates the Sertoli cell TJ barrier function, these cells were immunostained for different BTB marker proteins including a visualization of the F-actin network **(** **Fig. 5D** **)**. Importantly, the efficacy of the Rai14 knockdown was further confirmed when Sertoli cells transfected with Rai14 siRNA duplexes *versus* the non-targeting siRNA control duplexes were immunostained for Rai14, in which Rai14 staining in the actin filaments was considerably subdued in Rai14-silenced Sertoli cells *versus* control cells **(** **Fig. 5D** **)**. Interestingly, actin filaments in the Sertoli cells transfected with siRNA duplexes were dis-organized when compared to cells transfected with the non-targeting siRNA duplexes, since actin filaments in the Rai14 silenced cells were found to be truncated and defragmented, and some actin filaments became clustered but disorganized at the cell-cell interface **(** **Fig. 5D** **)**. Additionally, β-catenin and α-catenin, adaptor proteins that binds to the basal ES protein N-cadherin, but also associates with TJ-adaptor protein ZO-1 and interacts with TJ-protein occludin was found to be mis-localized in the Sertoli cells following Rai14 knockdown, in which both β- and α-catenin no longer restricted to the cell-cell interface, instead, catenins were internalized and moved into the cell cytosol **(** **Fig. 5D** **)**. These changes thus destabilized the Sertoli cell BTB barrier, leading to a disruption of the TJ-permeability barrier **(** **Fig. 5C, D** **)**.

### Knockdown of Rai14 in the testis *in vivo* impeded spermatid movement and polarity

**Figure 6:**
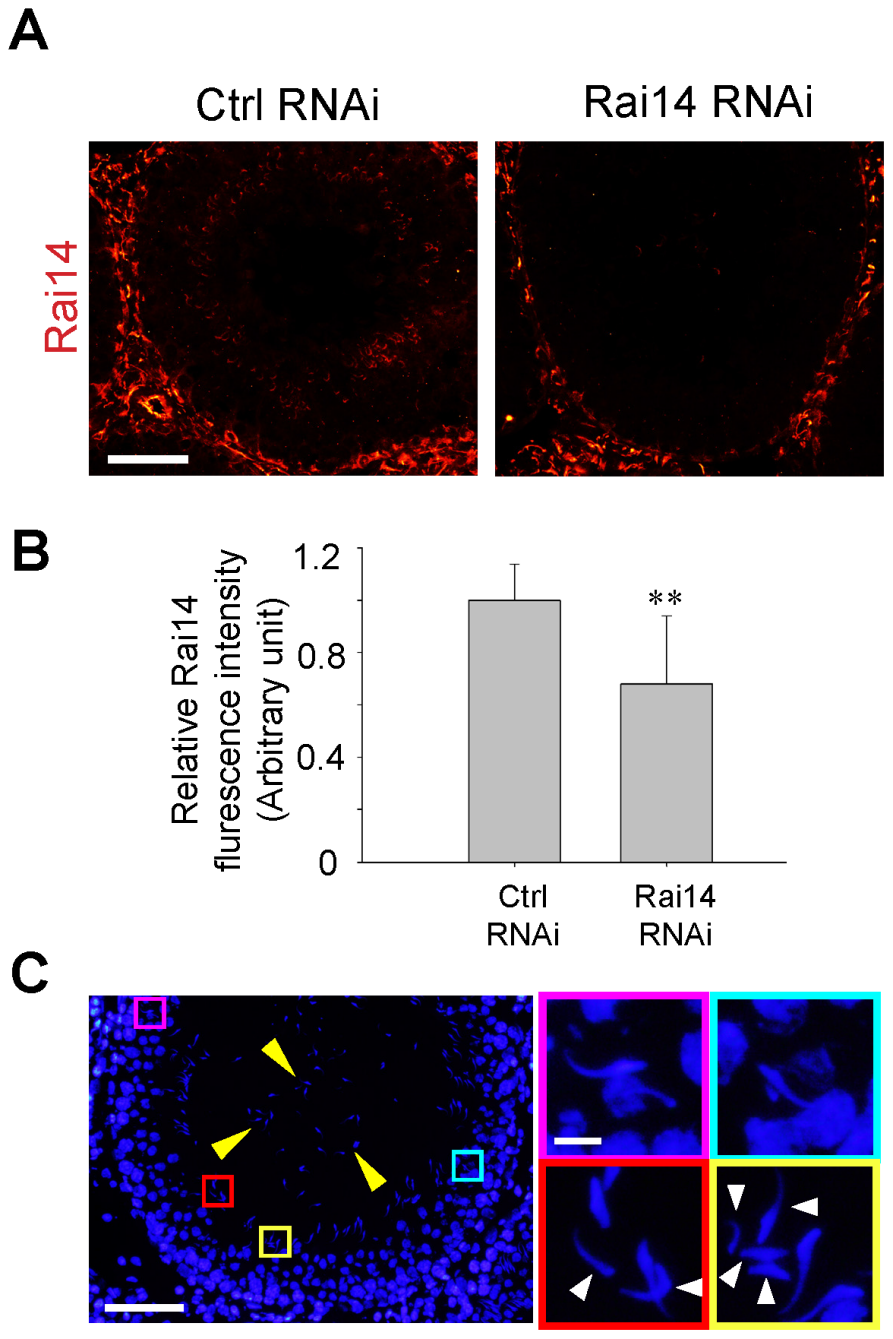
Effects of Rai14 knockdown in the testis *in vivo* on the spermatid polarity. (**A**) Rat testes were transfected with Rai14 specific siRNA duplexes (Rai14 RNAi) *vs.* control duplexes (Ctrl RNAi) to knockdown Rai14. A considerable decline in the Rai14 signals in the seminiferous epithelium in this late stage VII tubule, in particular at the apical ES, was noted as shown in (**B**) with ∼30% knockdown. Each bar in (B) is a mean±SD of *n* = 80 tubules at stage VII–VIII randomly selected from 4 rats. **, *P*<0.01. It is noted that Rai14 is an actin cross-linking and bundling protein, and actin filament bundles are the major constitute component of the apical ES, and apical ES is crucial to confer spermatid polarity and adhesion, the findings shown in (**C**) are consistent with the function of Rai14 at the apical ES. Following the knockdown of Rai14 in the testis, spermatids were detected in the tubule lumen of this stage VII tubule as a result of premature release of spermatids due to defects in the apical ES adhesion function (see “white” arrowheads). Furthermore, some spermatids were embedded in the epithelium (see “purple” and “blue” boxed areas) that failed to be transported to near the tubule lumen to prepare for spermiation at late stage VIII of the cycle. Additionally, spermatids that displayed a loss of polarity were seen (see “red” and “yellow” boxed areas and mis-oriented spermatids were annotated by “white” arrowheads). Scale bar = 100 µm in (**A**), 100 µm in (**C**), and 10 µm in inset in (**C**), which apply to corresponding micrographs in the same panel.

**Figure 7:**
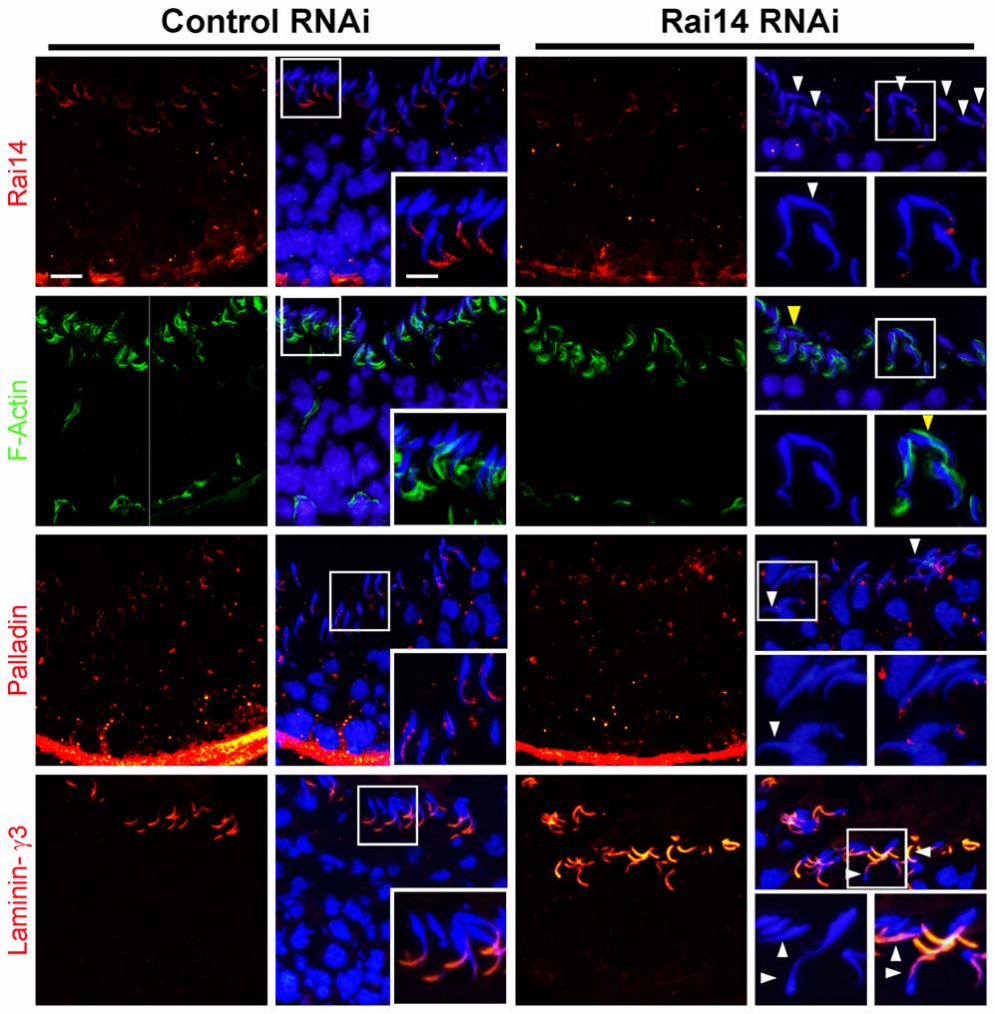
Effects of Rai14 knockdown in the testis on F-actin organization and protein distribution at the apical ES. Following a knockdown of Rai14 in the testis, a considerable loss of Rai14 signals (red fluorescence) were detected in the seminiferous epithelium of stage VII tubules as illustrated in this tubule here, in particular at the apical ES site. As shown in the Rai14 RNAi stage VII tubule, Rai14 was virtually not detected at the apical ES surrounding the heads of these step 19 spermatids, and polarity was disrupted in many of these spermatids lacking Rai14 since mis-oriented spermatids no longer pointing toward the basement membrane (see “white” arrowheads), perhaps due to the loss of actin filament bundles at the apical ES, which likely required Rai14 for their maintenance. This notion was strengthened when F-actin (green fluorescence) was visualized by FITC-conjugated phalloidin in Rai14 RNAi *vs.* control group. In control testes, at stage VII, F-actin was restricted to the tip of the spermatid head, mostly to the concave side of the head. After Rai14 knockdown, F-actin no longer restricted to the concave side of the spermatid head, instead considerable F-actin in many step 19 spermatids was found on the convex side of the spermatid head (see “yellow” arrowheads). This mis-localization of F-actin suggests that actin filament bundles in these spermatids were disrupted. Furthermore, while the signals of palladin (red fluorescence, an actin cross-linking and bundling protein), in the seminiferous epithelium in Rai14 knockdown group were not diminished *vs.* the control group, palladin was also found to become mis-localized, no longer found at the tips of the spermatid heads, but mis-localized and diffused away from spermatid head. Interestingly, the localization of laminin-γ3 chain (red fluorescence), an apical ES component of the integrin-laminin adhesion complex at the apical ES, was found not to be disrupted including mis-oriented spermatids with a loss of polarity. Scale bar = 20 µm, and scale bar in inset = 10 µm, which apply to other micrographs in the corresponding panel.

Based on the findings *in vitro* regarding the role of Rai14 on F-actin organization, such as at the basal ES at the Sertoli cell BTB **(** **Fig. 5** **)**, we sought to explore its functional significance at the apical ES since Rai14 was strongly expressed at this site in stage VII-VIII tubules **(see** **Figs. 2** **and** **3** **)**. When the testis was transfected with Rai14-specific siRNA duplexes *versus* the non-targeting control duplexes *in vivo*, Rai14 was found to be silenced by at least 30% when Rai14 fluorescence signals in the seminiferous epithelium of stage VII-VIII tubules randomly selected from 4 rats were quantified **(** **Fig. 6A, B** **)**. This was done because Rai14 was highly expressed only in VII-VIII tubules **(see** **Fig. 2** **)**. We attempted to quantify the Rai14 knockdown in the testis *in vivo* by immunoblotting as shown in **Fig. 5** for the *in vitro* studies, but these findings were not satisfactory since stage VII-VIII tubules constituted only ∼27% of all the tubules in the rat testis [52]; and amongst these, only ∼50% of the VII-VIII tubules displayed signs of Rai14 knockdown and in these tubules, only ∼20% of tubules displayed defects in spermatid polarity and spermatid transport **(see** **Table 3** **)** due to the efficacy of the knockdown using siRNA duplexes for transfection *in vivo*, based on multiple pilot experiments to optimize the transfection efficacy. Nonetheless, the data that were shown herein were summarized from those tubules in which Rai14 fluorescence signals were significantly reduced **(** **Fig. 6A, B** **)**; and in these tubules, Rai14 expression at the apical ES was considerably diminished and virtually undetectable after its knockdown *in vivo versus* the basal ES, the tunica propria and cells (*e.g*., endothelial cells of the microvessels and Leydig cells) in the interstitium **(** **Fig. 6A, B** **)**. More important, the *in vivo* knockdown of Rai14 was associated with a loss of spermatid adhesion and a loss of spermatid polarity **(** **Fig. 6C** **)**. These observations were further expanded by focusing on stage VI-VIII tubules when Rai14 expression at the apical ES was detectable **(** **Table 3** **)**. Consistent with the findings *in vitro* following its knockdown in Sertoli cells that impeded F-actin organization in these cells **(** **Fig. 6** **)**, and also the likely role of Rai14 on the actin filament bundles at the apical ES based on its spatiotemporal expression in the seminiferous epithelium during the epithelial cycle **(** **Figs. 2** **,** **3** **,** **4** **)**, a knockdown of Rai14 *in vivo* indeed was found to impede spermatid polarity and adhesion/transport **(** **Table 3** **)**. Following the knockdown of Rai14 in the testis, the Rai14 signals in the seminiferous epithelium of a stage VII tubule was considerably diminished **(** **Fig. 7** **)**. This loss of Rai14 at the apical ES was shown to associate with a mis-localization of F-actin and also actin cross-linker palladin at the apical ES **(** **Fig. 7** **)**. These changes thus caused the loss of spermatid polarity and adhesion, thereby perturbing spermatid transport that led to a defect in spermiation, illustrating changes in the organization of actin filament bundles at the apical ES can perturb apical ES function **(** **Fig. 7** **)**. Interestingly, the localization of laminin-γ3 chain at the apical ES was apparently unaffected even in mis-oriented spermatids **(** **Fig. 7** **)**.

## Discussion

In the early 1970s, the actin-filament bundles were first shown to be the core structure of the Sertoli cell BTB in rodent testes, which were sandwiched in-between cisternae of endoplasmic reticulum and the apposing plasma membrane of the adjacent Sertoli cells [53], [54]. The name ectoplasmic specialization (ES) was later coined to designate this ultrastructure at the Sertoli cell-elongating/elongated spermatid (step 8–19 spermatids in the rat testis) interface the apical ES [55], whereas the ES at the Sertoli-Sertoli cell interface at the BTB is known as the basal ES [8], [10]–[12], [56], [57]. While these actin filament bundles are crucial to confer the unique adhesive function and cell polarity to spermatids and Sertoli cells [14], [58], their regulation during the epithelial cycle remains virtually unknown. For instance, these actin filament bundles must be cyclically “de-bundled” and then “re-bundled” to facilitate the transport of elongating spermatids across the epithelium during spermiogenesis, involving actin cross-linking, bundling, polymerization, nucleation, cleavage, de-bundling and re-bundling. It is now known that the stage-specific spatiotemporal expression of the actin barbed end capping and bundling protein Eps8 [20], the branched actin polymerization inducing protein Arp2/3 complex [21], the Arp2/3-binding protein drebrin E [22], the Par-based protein Par6 [59], the actin filament cross-linking/bundling protein filamin A [23], the Scribble/Lgl/Dlg complex [40], the non-receptor protein kinase c-Yes [60] and the endocytic vesicle severing protein dynamin II [51] at the apical and/or basal ES during the epithelial cycle are involved in the organization of the actin filament bundles at the ES [7], [24]. Herein, we have shown that Rai14 is a crucial component of this growing list of actin binding/regulatory proteins that are involved in the organization and re-organization of the actin filament bundles at the ES.

Consistent with the earlier findings that Rai14 is an actin binding protein in the liver [1], we have demonstrated that Rai14 structurally interacts with actin in the rat testis. Interestingly, Arp3, Eps8, and drebrin E [20]–[22] were not the binding partners of Rai14. Instead, palladin, an actin cross-linking protein, was found to be structurally associated with Rai14. Since palladin was shown to interact with Eps8 in regulating F-actin dynamics [61], thus, even though Rai14 does not structurally interact with Eps8, it can still mediate its effects to confer actin filament bundles at the ES via its interaction with palladin. This possibility is supported by the findings reported herein that a knockdown of Rai14 in Sertoli cells *in vitro* leads to a disruption of the actin filament network in the Sertoli cell cytosol, impeding protein distribution at the Sertoli cell-cell interface (*e.g*., adaptor proteins α- and β-catenin), thereby perturbing the Sertoli cell TJ-permeability barrier. More important, its knockdown *in vivo* was shown to induce loss of spermatid polarity and adhesion, such that the head of elongating/elongated spermatids no longer pointing toward the basement membrane, and spermatids became entrapped in the seminiferous epithelium that led to defects in spermiation due to the lack of proper F-actin re-organization to confer proper spermatid transport across the epithelium during the epithelial cycle. These findings strongly suggest that the apical ES function was compromised following the knockdown of Rai14, in which the integrity and/or the dynamics of the actin filament bundles were perturbed, since this ultrastructure is known to be crucial to confer spermatid polarity and adhesion [8], [9], [24], [62]. Indeed, studies by F-actin staining have demonstrated a mis-localization of F-actin at the apical ES following Rai14 knockdown. Thus, Rai14 may serve as a novel protein that recruits other regulatory proteins to the apical ES to facilitate changes in cell shape and the relative position of developing spermatids in the epithelium during spermiogenesis. This possibility is also supported by the stage-specific spatiotemporal expression of Rai14 at the apical ES during the epithelial cycle in which the localization of Rai14 at the apical ES shifts continuously during spermiogenesis. For instance, Rai14 was restricted mostly to the concave side of the tip of the step 19 spermatid head in stage VII tubules, but it shifted to the basal region of the spermatid head at stage VIII, apparently being used to facilitate the alignment of spermatids at the edge of the tubule lumen to prepare for spermiation.

Based on the findings reported herein, which coupled with current concepts in the field, Rai14 along with other regulators in the list described above may regulate ES function via their effects on the actin filament bundles utilizing the following mechanical pathway. The loss of Rai14, such as illustrated in studies by its silencing using RNAi that perturbs the actin filament organization in Sertoli cells at the BTB *in vitro*, well as at the apical ES *in vivo* in stage VII–VIII tubules; or at late stage VIII of the epithelial cycle when the expression of Rai14 was low at the apical and the basal ES, may destabilize the ES. This, in turn, facilitates endocytic vesicle-mediated protein trafficking, such as protein endocytosis, transcytosis and recycling. This possibility is supported by recent findings that at this stage of the epithelial cycle, protein endocytosis rapidly occur at the concave side of the spermatid head, forming a giant endocytic vesicle-like ultrastructure formerly designated apical tubulobulbar complex [63]–[66]. In short, the transient down-regulation of Rai14 at stage VIII of the epithelial cycle that promotes defragmentation and truncation of the actin filament bundles at the ES may facilitate protein endocytosis, so that the transient loss of adhesion protein complexes at the apical or the basal ES destabilizes cell adhesion function at these sites, promoting the release of mature spermatids at spermiation or BTB restructuring, which take place at late stage VIII of the cycle. It is logical to speculate that Rai14 is not working alone, instead, it exerts its effects in concert with other actin regulatory proteins (*e.g*., Eps8, Arp2/3 complex, drebrin E, Par6, Scribble, Dlg, Lgl, filamin A) at these sites so that actin filament bundles at the ES can be precisely regulated during the epithelial cycle to facilitate internalization, transcytosis and recycling of adhesion protein complexes at the apical (*e.g*., integrin-laminin, nectin-afadin, JAM-C-ZO-1) or the basal ES (*e.g*., occludin-ZO-1, claudin-ZO-1, JAM-A-ZO-1, N-cadherin-β-catenin). For instance, as shown herein in the *in vivo* silencing experiments, following a knockdown of Rai14, while it did not impede the expression of actin cross-linking protein palladin which was shown to be an interacting partner of Rai14, it did induce mis-localization of palladin at the apical ES, facilitating a mis-organization of F-actin at the apical ES. This concept is also supported by studies in other epithelia in which organization and re-organization of F-actin in epithelial cells regulate endocytic vesicle-mediated protein trafficking [67]–[71]. Furthermore, this event is physiologically necessary since spermatids are metabolically quiescent cells, however, the apical ES is continuously needed to anchor newly arise step 8–19 spermatids from spermiogenesis, and since there is a fixed number of Sertoli cells in adult mammalian testes [33] at a Sertoli:germ cell ratio of ∼1∶30–1∶50 [72], it is physiologically not possible that *de novo* synthesis of ES proteins is the only mechanism to replenish necessary structural proteins during spermiogenesis or BTB restructuring during the epithelial cycle. If proteins at the “old” BTB or “degenerating” apical ES site can be internalized to facilitate ES restructuring, and they can be transcytosed and recycled to establish “new” BTB or apical ES, resources in the seminiferous epithelium can be better utilized to safe guard the cellular homeostasis in the epithelium. This concept has recently been shown using time-lapsed microscopy with claudin-3 as a marker in which this TJ-integral membrane protein was being “recycled” from the basal to the apical region of preleptotene spermatocytes in transit at the BTB [73]. It is highly possible that some of the “aged” ES proteins can be targeted to degradation via either the endosome or the ubiquitin pathway since endocytic-mediated protein degradation has been shown to occur in Sertoli cells [5], [6]. Nonetheless, studies are needed to understand the underlying mechanism(s) by which (or the involving biomolecules) endocytosed proteins are selected for transcytosis and recycling *versus* endosome/ubiquitin-mediated degradation.
